# Probiotic supplementation to mitigate berotralstat gastrointestinal side effects: a multicenter case series from the ITACA network

**DOI:** 10.3389/fimmu.2026.1790199

**Published:** 2026-04-29

**Authors:** Antonio Gidaro, Maria Domenica Guarino, Francesco Arcoleo, Donatella Bignardi, Cristian D’Antonio, Luisa Brussino, Pietro Accardo, Stefania Nicola, Stefano Pucci, Miriam Cognigni, Valentina Popescu Janu, Chiara Cogliati, Emanuele Bizzi, Mauro Cancian

**Affiliations:** 1 Internal Medicine, L. Sacco Hospital, Azienda Socio Sanitaria Territoriale (ASST) Fbf-Sacco, Milan, Italy; 2 Allergy Unit, Hospital of Civitanova Marche, Civitanova Marche, Italy; 3 Azienda Ospedaliera (AO) “Ospedali Riuniti Villa Sofia-Cervello”-Presidio Ospedaliero Cervello, Palermo, Italy; 4 Department Allergy Unit, Istituti di Ricovero e Cura a Carattere Scientifico (IRCCS) Ospedale Policlinico San Martino, Genova, Italy; 5 Allergy Unit, Santissima (SS) Trinita Borgomanero Hospital, Azienda Sanitaria Locale Novara (ASL NO), Novara, Italy; 6 Allergy and Immunology Unit, Department of Medical Science, University of Turin, Mauriziano Hospital, Turin, Italy; 7 Internal Medicine, Vita-Salute University San Raffaele, Milan, Italy; 8 Department of Systems Medicine, University Hospital of Padua, Padua, Italy

**Keywords:** berotralstat, gastrointestinal side effects, hereditary angioedema, ITACA, long term prophylaxis, microbiome, probiotics

## Abstract

**Introduction:**

Berotralstat, the first oral plasma kallikrein inhibitor approved for hereditary angioedema (HAE) prophylaxis, may be associated with gastrointestinal (GI) side effects, particularly during the first three months of therapy. Probiotics have been shown to reduce GI disturbances in several conditions. This pilot study described GI tolerability in patients receiving initiation-phase probiotic co-administration alongside berotralstat and explored whether this supportive strategy merits further controlled evaluation.

**Materials and methods:**

We analyzed 25 adolescents and adults with HAE treated with berotralstat across six Italian centers (December 2023-November 2025). All patients received probiotics during the early treatment phase. Demographic and clinical data, side effects, and monthly HAE attack rates were collected. Severity of complaints was graded using the Common Terminology Criteria for Adverse Events (CTCAE).

**Results:**

Participants were 60% females, and the mean age for the cohort was 45 years (range 12-82). The most common probiotics were *Lacteol^®^*, *Codex^®^*, and *Lactoflorene Plus^®^*. GI complaints occurred in 5/25 patients (20%); only 3/25 (12%) experienced GI side effects while receiving probiotics. 2/25 GI complaints occurred after probiotic discontinuation. No serious side effects were reported. Mean monthly attack rate decreased from 2.6 to 0.8 attacks per month, ~3.3-fold reduction from baseline.

**Conclusion:**

Probiotic co-administration during early berotralstat therapy was accompanied by a low incidence of GI side effects, while clinical effectiveness was maintained. These preliminary findings support further controlled studies to validate probiotics as a supportive strategy for improving the tolerability of berotralstat.

## 1 Introduction

Hereditary angioedema caused by the C1-inhibitor deficiency (HAE-C1INH) is a rare genetic disorder characterized by recurrent attacks of subcutaneous and submucosal swelling involving the skin, gastrointestinal tract, and upper airways (1, 2). It affects approximately 1–2 individuals per 100,000 worldwide, and diagnosis is often delayed or missed (3). Because HAE is a chronic, lifelong disease, there is a continuous need for long-term prophylactic options that are effective, well-tolerated, and impose a minimal treatment burden (4).

Long-term prophylaxis (LTP) has been shown to improve disease control and quality of life compared with on-demand treatment alone, reducing anxiety, limitations in daily activities, and absenteeism (5). Current LTP options include both oral and parenteral therapies. Parenteral administration may result in a significant treatment burden, particularly for children, adolescents, and patients with needle phobia or poor venous access. Historically, oral prophylaxis relied on attenuated androgens and tranexamic acid; however, due to limited efficacy, safety concerns, drug interactions, and contraindications, androgens are currently recommended by the most recent WAO/EAACI guidelines as second-line options in settings where modern, HAE-specific therapies are unavailable, and tranexamic acid is not recommended because data for its efficacy are largely lacking (4, 6, 7).

Among newer therapeutic options, berotralstat is the first oral plasma kallikrein inhibitor approved for routine prevention of recurrent HAE attacks in adults and adolescents aged 12 years and older, with a pediatric extension to children aged 2 to 12 years under regulatory evaluation (8–12). Despite its advantages in convenience and adherence, gastrointestinal (GI) side effects are frequently reported (10, 13–15). Although these events are typically mild and transient, and can often be mitigated by taking the medication with food, they may negatively affect treatment satisfaction and lead to early discontinuation, as reported in APEX-S (14). The underlying mechanisms of berotralstat-induced GI side effects remain unclear, and no specific strategies have been formally evaluated to prevent them.

Emerging evidence indicates alterations in the microbiome in HAE-C1INH, including reduced richness and compositional shifts in patients with recent abdominal attacks and oropharyngeal dysbiosis in those with recent laryngeal edema (16, 17). Thus, many patients initiate prophylaxis with a background of GI vulnerability related to disease biology, which may explain why berotralstat-related GI complaints tend to cluster early and attenuate over time. In APeX-2 (Study 302, Part 1), at the recommended dose of 150 mg/day, the most common GI side effects were abdominal pain (23%), vomiting (15%), and diarrhea (15%); these symptoms typically occurred soon after initiation and self-resolved. An earlier analysis showed GI side effects peaking in the first month and declining thereafter, and pharmacovigilance data indicate that GI side effects account for ~47% of reported reactions. Taken together, these observations may suggest a temporal sequence: pre-existing microbiome imbalance in some patients and an early, time-limited “risk window” for GI complaints after starting berotralstat.

Probiotics have shown cross-indication benefit for drug-related GI syndromes, most notably antibiotic-associated diarrhea (RR 0.58; NNT~13) and in primary-care guidance for common GI conditions, often using *Lactobacillus rhamnosus* GG or *Saccharomyces boulardii* at 10^9^–10^10^ CFU/day (18, 19). Additional syntheses report the mitigation of chemotherapy-induced diarrhea and improved tolerability of metformin (20, 21). In HAE-C1INH, disease-specific data link microbiome composition with attack phenotypes in the gut and oropharynx (16, 17). Taken together, these observations motivate a short, initiation-phase probiotic administration to enhance GI tolerability when starting berotralstat as a pragmatic strategy to preserve adherence. By supporting mucosal barrier integrity, modulating luminal metabolites and inflammatory tone, and competitively limiting opportunistic taxa, a short, time-limited probiotic course could plausibly improve GI tolerability without interfering with kallikrein inhibition. Despite this biologic rationale and cross-indication evidence for GI symptom mitigation, no studies have directly evaluated the co-administration of probiotics with berotralstat in HAE. Current clinical practice largely relies on counseling about the transient nature of these events and simple measures such as taking the drug with food, which may be insufficient for a subset of patients at the highest risk of early discontinuation.

To address this gap, we present a longitudinal, real-world, multicenter case series of patients with HAE who initiated (or switched to) berotralstat while receiving concomitant probiotic therapy during the early treatment phase. Our objectives were to (*i*) describe the incidence, timing, and severity of GI complaints under concomitant use, (*ii*) explore whether events differed when probiotics were on board vs. discontinued, and (*iii*) assess whether berotralstat’s clinical effectiveness (monthly attack rate) was maintained in this context. These hypothesis-generating data aim to inform the design of prospective, controlled evaluations of initiation-phase probiotic co-administration as a simple, low-burden strategy to enhance tolerability and persistence with oral prophylaxis in HAE.

## 2 Materials and methods

In this longitudinal study, we collected data from 25 adolescents and adults with hereditary angioedema (HAE) who initiated berotralstat treatment between December 2023 and November 2025 in six reference centers of the Italian Network for Hereditary and Acquired Angioedema (ITACA). All patients received probiotic supplementation during the early phase (initiation) of berotralstat therapy. No contemporaneous control group of berotralstat-treated patients without probiotic co-administration was included. Baseline demographic and clinical characteristics, prior prophylactic treatments, durations of berotralstat and probiotic use, monthly HAE attack rate, and side effects were recorded. Adverse events were collected from routine clinical documentation at participating centers. Event-level information on severity, timing relative to probiotic exposure, treatment interruption, and duration was available. Patients were followed up for at least 1 month. No predefined follow-up period was established. Because this was a real-world registry-based case series, berotralstat adherence/compliance was not formally assessed by pill counts, prescription refill data, or standardized patient-reported measures. Probiotic adherence/compliance was also not formally assessed. The decision to initiate, continue, discontinue, or switch probiotic therapy, as well as the choice of product and duration of use, was made according to routine clinical practice at each center and was not standardized across sites. Enrollment in the ITACA Registry was approved by the ethics committee of the coordinating center (Comitato etico Milano area 1, Italy) on 5 May 2017. According to the Ethics Committee, all patients signed written informed consent for the use of their anonymized data.

The following eligibility criteria were considered. Inclusion: (*i*) confirmed HAE due to C1-inhibitor deficiency or dysfunction (HAE-C1INH; types I/II); (*ii*) age ≥12 years; (*iii*) start of berotralstat within the study window; (*iv*) receipt of any probiotic during the initiation phase, defined as probiotic use started within the first 30 days of berotralstat initiation. Exclusion: (*i*) age <12 years; (*ii*) angioedema different from HAE-C1INH (e.g., HAE with normal C1-INH, or acquired angioedema); (*iii*) concurrent GI disorders unrelated to HAE judged likely to confound GI symptom attribution (e.g., active inflammatory bowel disease, chronic infectious diarrhea, uncontrolled celiac disease).

The primary outcome was the incidence of GI side effects during the co-administration period of berotralstat and probiotics. The severity of side effects was assessed using the Common Terminology Criteria for Adverse Events (CTCAE). Secondary outcomes included the monthly HAE attack rate and the change in attack rate before berotralstat initiation compared with the last available follow-up, to assess whether berotralstat maintained its effectiveness in patients receiving probiotics.

For each patient, the on-treatment monthly attack rate was calculated from the berotralstat start to the last available follow-up. Monthly rates were normalized to a 30-day month as:


$$\text{Monthly attack rate}=\frac{\text{number of attacks in the interval}}{\text{number of days in the interval}}\times 30$$

The baseline monthly attack rate corresponded to the pre-treatment monthly attack rate recorded in routine clinical documentation before berotralstat initiation; no standardized pre-treatment observation window was mandated.

Furthermore, attack-free days were defined for each patient as in the following formula and were reported in the text:


$$\text{\% attack‐free}=\frac{\text{days without attacks}}{\text{total on‐treatment days}}\times 100$$

Patients with 0 attacks during the on-treatment observation window were classified as attack-free; those with ≤1 attack/month met a low-activity threshold used for descriptive purposes.

Each attack was categorized, when documentation allowed, as abdominal, peripheral, face/oral cavity (head/neck/oropharyngeal, including laryngeal when specified), or mixed (multi-site within the same episode). Site-specific proportions were reported as % attacks in each category.

Descriptive statistics were used, and results are presented as means with standard deviations. No formal statistical comparisons were made. For the monthly attack rate, we additionally report as descriptive estimates the mean ± 95% confidence interval and median ± 95% confidence interval (CI), calculated as 1.57·IQR/√n, where IQR is the interquartile range based on the notched-boxplot approximation described by McGill et al. (22).

## 3 Results

Demographic and baseline treatment characteristics are summarized in **Table 1**. In brief, 25 patients were enrolled (male 10/25, 40%; female 15/25, 60%), with a median age of 48 years (range 12-82), a median age at diagnosis was 23 years (range 2-76), and a median weight of 70 kg (range 50-109). Age distribution by predefined bands was as follows: 12–17 years, 3/25 (12%); 18–44 years, 9/25 (36%); 45–64 years, 10/25 (40%); and ≥65 years, 3/25 (12%). Sixteen patients (64%) had received prior prophylaxis, most commonly danazol (7/25, 28%), followed by lanadelumab (3/25, 12%), plasma-derived C1INH. (pdC1INH; 4/25, 16%; 3/25 s.c. and 1/25 i.v.), deucrictibant (1/25, 5%), and tranexamic acid (1/25, 4%); 10 patients (40%) had never received long-term prophylaxis before starting berotralstat. The mean monthly attack rate at baseline was 2.6 (95% CI: 1.7; 3.4).

**Table 1:** Population Characteristics.

N	25	Notes
Male. n (%)	10 (40%)	
Female, n (%)	15 (60%)	
Mean Age, years (± SD)	45.0 (19.1)	
Median Age, years (range)	48 (12-82)	
Age bands, n (%)	12-17, 3 (12%) 18-44, 9 (36%) 45-64, 10 (40%) ≥65, 3 (12%)	
Mean age at diagnosis, years (± SD)	24.8 (16.5)	
Median age at diagnosis, years (range)	23 (2-76)	
Mean Weight, kg (± SD)	71.4 (15.7)	
Median Weight, kg (range)	70 (50-109)	
Previous prophylaxis, n (%)	16 (64%)	
Danazol, n (%)	7 (28%)	
Lanadelumab, n (%)	3 (12%)	
C1-INH, n (%)	4 (16%)	
Deucrictibant, n (%)	1 (4%)	
Tranexamic acid, n (%)	1 (4%)	
No treatment, n (%)	9 (36%)	
Average monthly attack, n (± SD)	2.6 (2.2)	
Median monthly attack, days (range)	3 (0-8)	
Average duration of berotralstat treatment, days (± SD)	172.6 (157.2)	
Median duration of berotralstat treatment (IQR)	111 (52-255)	
Average duration of probiotic treatment, days (± SD)	34.0 (52.1)	
Median duration of probiotic treatment, days (IQR)	16 (8-31)	
Probiotic used, n (%)		*Species*
Lacteol^®^	10 (40%)	*Lactobacillus fermentum, Lactobacillus delbrueckii*
Codex^®^	5 (20%)	*Saccharomyces boulardii*
Lactoflorene plus^®^	4 (16%)	*Lactobacillus acidophilus; Bifidobacterium animalis; Bifidobacter coagulans: Lactobacillus paracasei*
Enterolactis plus 24 billion^®^	2 (8%)	*Lactobacillus casei*
Reuterin gg^®^	1 (4%)	*Lactobacillus reuteri; Lactobacillus rhamnosus*
Reuflor^®^	1 (4%)	*Lactobacillus reuteri*
Zirfos^®^	1 (4%)	*Bifidobacterium longum*
Bifilact^®^	1 (4%)	*Bifidobacterium lactis; Lactobacillus acidophilus; Lactobacillus plantarum; Lactobacillus paracasei*

IQR, range interquartile; pdC1-INH, plasma derived C1-esterase inhibitor.

The probiotic product mix was heterogeneous (**Table 1**). The most frequently used preparations were Lacteol^®^ (*Lactobacillus fermentum* + *Lactobacillus delbrueckii*) in 10/25 patients (40%), Codex^®^ (*Saccharomyces boulardii*) in 5/25 (20%), and Lactoflorene Plus^®^ (multi-strain blend including *Lactobacillus* spp. and *Bifidobacterium* spp.) in 4/25(16%). Less commonly used products were Enterolactis Plus 24 billion^®^ (*Lactobacillus casei*) 2/25 (8%), Reuterin gg^®^ (*Lactobacillus reuteri* + *Lactobacillus rhamnosus*) 1/25 (4%), Reuflor^®^ (*Lactobacillus reuteri*) 1/25 (4%), Zirfos^®^ (*Bifidobacterium longum*) 1/25 (4%), and Bifilact^®^ (multi-strain *Lactobacillus/Bifidobacterium* blend) 1/25 (4%). Overall, multi-strain formulations accounted for 16/25 (64%) courses, whereas single-strain products accounted for 9/25 (36%). Most regimens contained at least one *Lactobacillus* species (19/25, 76%), and *S. boulardii* monotherapy was used in 5/25 (20%).

At the time of analysis, the mean ± SD duration of berotralstat treatment was 172.6 ± 157.2 days (median 111 days; interquartile range [IQR] 52–255 days), while the mean probiotic co-administration lasted 34.0 ± 52.1 days (median 16 days; IQR 8–31 days). The short courses of berotralstat and probiotic treatments were due to treatment interruption. Probiotic exposure was mainly concentrated in the initiation phase, aligning with the period when side effects most commonly occur: 17/25 (68%) received a ≤ 30-day course, 5/25 (20%) received 31–90 days, and 3/25 (12%) received >90 days.

During the follow-up period, 20/25 (80%) patients reported no GI complaints. Only 3/25 (12%) experienced GI side effects during berotralstat + probiotic therapy, while an additional 2/25 (8%) patients developed symptoms only after probiotic interruption. The post-discontinuation cases involved *Lactobacillus reuteri-*based products: one patient reported nausea (mild) with headache (mild) after stopping Reuterin gg^®^, and another had diarrhea (moderate) after stopping Reuflor^®^. Among on-treatment events, retrosternal pyrosis (moderate) occurred with Lactoflorene Plus^®^ and led to discontinuation of berotralstat (1/25, 4%), while vomiting (moderate) occurred with Lacteol^®^ and abdominal pain (moderate) occurred with Codex^®^. No serious adverse events were observed. Duration information derived from routine clinical documentation was available for all recorded events. The longest durations were observed in the two cases reported after probiotic interruption (nausea/headache, 90 days; diarrhea, 90 days), whereas events occurring during concomitant berotralstat + probiotic therapy lasted 30 days(retrosternal pyrosis), 3 days (vomiting), and 1 day (abdominal pain). Full event terms, severity, and their timing relative to probiotic use are reported in **Table 2**.

**Table 2:** Description of adverse events observed.

Adverse event	Severity	Probiotics	*Species*	Details	Duration, days
Nausea	Mild	Reuterin gg^®^	*Lactobacillus reuteri; Lactobacillus rhamnosus*	After probiotic interruption	90
Headache	Mild	Reuterin gg^®^	*Lactobacillus reuteri; Lactobacillus rhamnosus*	After probiotic interruption	90
Retrosternal Pyrosis	Moderate	Lactoflorene plus^®^	*Lactobacillus acidophilus; Bifidobacterium animalis; Bifidobacter coagulans: Lactobacillus paracasei*	Led to berotralstat discontinuation	30
Diarrhea	Moderate	Reuflor^®^	*Lactobacillus reuteri*	After probiotic interruption	90
Vomiting	Moderate	Lacteol^®^	*Lactobacillus fermentum, Lactobacillus delbrueckii*		3
Abdominal Pain	Moderate	Codex^®^	*Saccharomyces boulardii*		1

GI, gastrointestinal.

**Figure 1:**
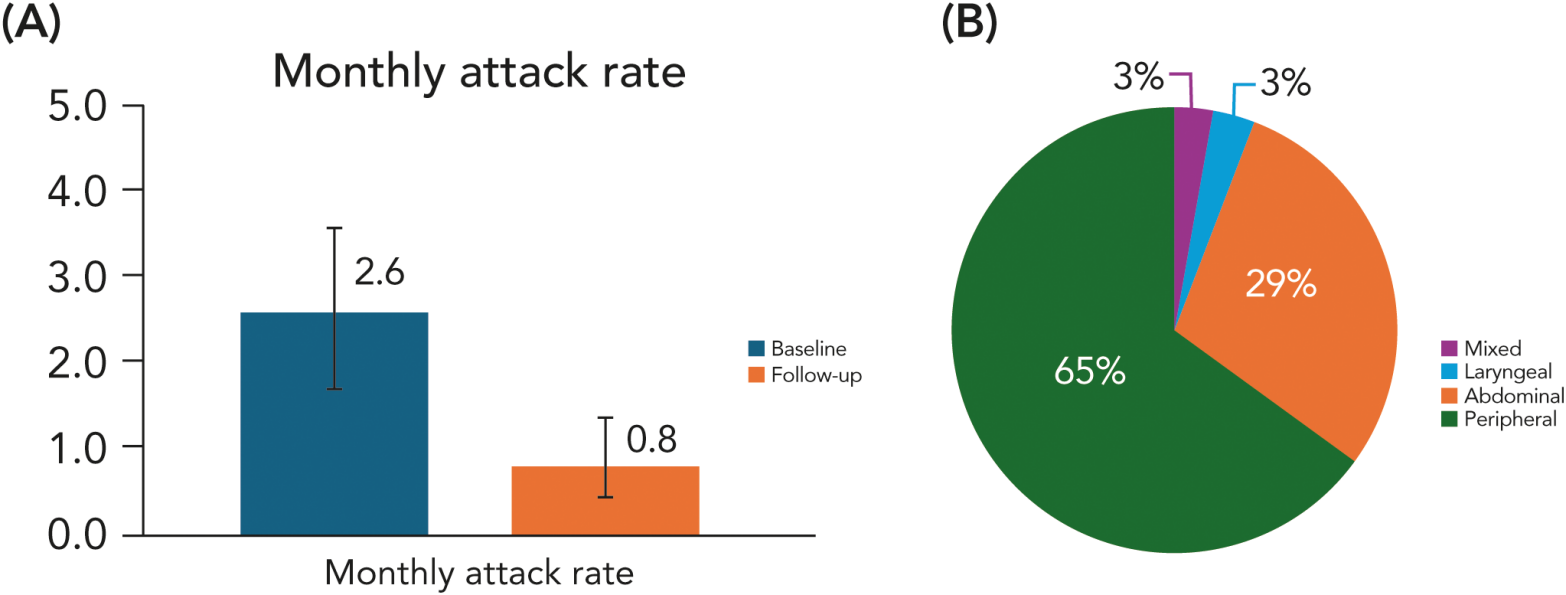
**(A)** Monthly HAE attack rate (95% CI) from baseline to last follow-up of the berotralstat treatment period. **(B)** Locations of attack recorded during the berotralstat treatment period.

Following the initiation of berotralstat treatment, the mean monthly attack rate decreased from 2.6 (95% CI 1.7-3.4) to 0.8 (CI 95%: 0.4; 1.2), representing a ~70% reduction from baseline (**Figure 1A**). Seven out of 25 (28%) patients were attack-free (monthly rate = 0), and 18/25 (72%) recorded ≤1 attack/month during the on-treatment observation window (from berotralstat start to last follow-up; median 111 days). Subgroup analyses were consistent with the overall findings: treatment-naïve patients (n=9), who had not received any long-term prophylaxis for HAE before starting berotralstat, improved from 4.11 to 0.33 attacks/month (-92.1%); 44.4% were attack-free, and 88.9% had ≤1 attack/month (median of follow-up exposure was 171 days). Patients with prior long-term prophylaxis (n=16) improved from 1.71 to 1.03 attacks/month (-39.6%); 18.8% were attack-free, and 62.5% had ≤1 attack/month (median of follow-up exposure was 101 days). Considering the total exposure time, the median monthly attack rate was 0.35 ± 0.33 attacks/month, indicating that most patients clustered at the lower end of attack frequency. Overall, 99% of the 4,287 cumulative on-treatment days with berotralstat were attack-free.

Across the study, 61 attacks were recorded, and the location of 31/61 (51%) was documented (**Figure 1B**). Among the 31 documented site-specific attacks, the distribution was peripheral (20/31; 65%), abdominal (9/31; 29%), laryngeal (1/31; 3%), and mixed (1/31; 3%). Among the 25 patients enrolled in the study, only 3 (12%) discontinued berotralstat treatment: 2 (8%) due to lack of efficacy and 1 (4%) due to gastrointestinal side effects. **Table 3** summarizes key outcomes.

**Table 3:** Tolerability, persistence, and effectiveness with probiotic co-administration at berotralstat initiation.

Outcome	Result	Notes
GI side effects (any)	5/25 (20%)	All mild-moderate; no serious events
GI side effects while on probiotics	3/25 (12%)	During concomitant berotralstat + probiotics
GI complaints after probiotic discontinuation	2/25 (8%)	Symptoms appeared only after stopping probiotics
On berotralstat at last follow-up (persistence)	22/25 (88%)	
Berotralstat discontinuation	3/25 (12%)	Due to lack of efficacy: 2/25 (8%) Due to GI complaint: 1/25 (4%)
Median monthly attack rate during berotralstat treatment (mean, CI 95%)	Baseline 2.6 (1.7; 3.4) Post-treatment 0.8 (0.4; 1.2)	~70%, ~3.3-fold reduction
Monthly attack rate during berotralstat treatment (median, 95%)	0.35 (0.02; 0.67)	At last follow-up
Attack-free at last follow-up	7/25 (28%)	Monthly rate = 0
≤1 attack/month	18/25 (72%)	

CI 95%, confidence interval at 95%, GI, gastrointestinal.

## 4 Discussion

In this multicenter real-world pilot study, co-administration of a probiotic during berotralstat initiation was accompanied by a low frequency of reported GI complaints and high treatment persistence, while clinical effectiveness was maintained. During the probiotic co-administration window, only 12% (3/25) of patients experienced GI complaints, and no serious adverse events occurred. Notably, 8% (2/25) developed new GI symptoms only after probiotics were discontinued, a temporal pattern of clinical interest within the early “risk window”. In addition, the longest documented event durations were observed in the cases reported after probiotic interruption rather than during concomitant probiotic exposure. However, given the small number of events and the possible bias of duration reported, this temporal pattern should be interpreted cautiously. These observations compare favorably with GI side effect rates reported in clinical trials and surveillance analysis (approximately 54% in APeX-S and 47% in APeX-2 over six months, ~40-49% in real-world cohorts, and ~47.5% of reports in a pharmacovigilance dataset), being compatible with a clinically meaningful indication for improved tolerability in our cohort (10, 11, 13–15, 23). However, such cross-study comparisons should be interpreted cautiously because of differences in study design, follow-up duration, patient selection, and adverse-event ascertainment. Accordingly, these external data provide context only and do not substitute for an internal control.

Effectiveness of berotralstat was preserved when administered with probiotics: the mean monthly attack rate declined from a baseline of 2.6 (95% CI, 1.7-3.4) to 0.8 (95% CI, 0.4-1.2), a ~70% reduction, with ~99% of on-treatment days attack-free, 28% of patients attack-free at last follow-up, and 72% at ≤1 attack/month. These efficacy data are reported to show that concomitant probiotic use did not appear to compromise the expected prophylactic effect of berotralstat, rather than to suggest any additive effect of probiotics on attack control. Berotralstat effectiveness aligns with reductions reported in pivotal randomized controlled trials and is directionally consistent with real-world datasets where probiotics were not administered (9, 10, 14, 15, 23). In this pilot study, treatment persistence was also encouraging: 12% (3/25) discontinued berotralstat overall (4% for GI complaints and 8% for lack of efficacy), a lower percentage compared to post-approval cohorts reporting ~33% discontinuation with 12% for side effects and 19% for perceived lack of effectiveness (23).

A biologically plausible rationale exists for a probiotic effect on early GI tolerability. Most products used contained *Lactobacillus* species, which are frequently prescribed for drug-related GI disturbances. Although the mechanisms underlying berotralstat-related GI complaints remain unclear, microbiome modulation is a reasonable hypothesis that merits prospective testing. Complementarily, emerging data on microbiome alterations in HAE-C1INH (including shifts in *Bifidobacterium* species), even in patients not receiving berotralstat, suggest a host-microbiome axis that could plausibly affect symptoms and tolerability (16, 17). In mast cell-mediated urticaria, a condition that frequently associates with angioedema, a recent metanalysis of 12 clinical trials reported a beneficial effect of co-administration of probiotics and antihistaminic agents not only in adverse event rate, but also in improving urticaria symptoms control and quality of life. Moreover, a reduction in serum interleukin and tumor necrosis factor (TNF) levels was observed (24). However, because no direct microbiome analyses were performed in the present study, this biologically plausible explanation could not be explored directly in our cohort.

Randomized controlled studies are needed to evaluate the effect of adding probiotics to prophylactic treatment, including in HAE, given this study’s significant limitations. The most important limitation is the absence of a contemporaneous control group not receiving probiotics, which precludes causal inference; nonetheless, the observed GI side-effect rate during co-administration was markedly lower than in the published literature (10, 13–15, 23). Additional limitations include a small sample size, heterogeneity in probiotic strain/dose/duration, variable follow-up, incomplete site documentation for some attacks, the absence of direct microbiome assessment (no stool sampling or microbiome profiling), and the absence of patient-reported outcome measures for quality of life and treatment satisfaction. Residual confounding related to prior long-term prophylaxis exposure, baseline HAE activity (including GI vulnerability), and concomitant medications cannot be excluded. No standardized washout period from prior long-term prophylaxis was mandated or systematically recorded before berotralstat initiation; therefore, residual pharmacodynamic effects of prior therapies, particularly lanadelumab and pdC1-INH, may have influenced early attack frequency and tolerability outcomes. Furthermore, the use of on-demand therapy during the observation period was not systematically recorded. Moreover, follow-up duration was not standardized and varied substantially across patients, which may have influenced both adverse-event capture and the estimation of on-treatment attack rates. Prospective controlled studies, ideally including a contemporaneous berotralstat-treated group without probiotics and complete adverse-event capture, are warranted to assess and further confirm whether initiation-phase probiotic supplementation improves gastrointestinal tolerability.

Concluding, in this multicenter, real-world case series, initiation-phase probiotic co-administration during berotralstat start-up was accompanied by a low frequency of GI complaints, high treatment persistence, and preserved clinical effectiveness in this cohort. These observations support that time-limited probiotic supplementation may be a pragmatic, low-burden adjunct to improve early gastrointestinal tolerability and reduce discontinuation risk when initiating berotralstat, an issue of clear relevance in routine care, where early GI complaints can undermine satisfaction and adherence. Because the intervention is inexpensive and generally well tolerated in immunocompetent patients, it appears feasible to implement it (*e.g.*, a standardized “starter” course for the first 8–12 weeks) in clinical practice without interfering with kallikrein inhibition. If corroborated, an “initiation-phase probiotic” approach could be incorporated into HAE care pathways as a simple supportive strategy to enhance tolerability, persistence, and overall patient experience with oral prophylaxis. It may be generalizable to other oral agents where early GI complaints limit long-term use. Future prospective studies should integrate serial stool sampling and microbiome profiling to determine whether changes in microbial composition or dynamics are associated with GI tolerability during berotralstat initiation. Future studies should also predefine the collection and stratified analysis of potential confounders, including prior prophylaxis exposure, baseline abdominal/GI disease activity, and concomitant medications. Finally, prospective studies should capture the frequency and type of on-demand therapy used during follow-up to better contextualize attack severity, disease control, and patient management.

## Data Availability

The raw data supporting the conclusions of this article will be made available by the authors, without undue reservation.
